# Design and Analysis of Cognitive Interviews for Comparative Multinational Testing

**DOI:** 10.1177/1525822X11414802

**Published:** 2011-10-17

**Authors:** Kristen Miller, Rory Fitzgerald, José-Luis Padilla, Stephanie Willson, Sally Widdop, Rachel Caspar, Martin Dimov, Michelle Gray, Cátia Nunes, Peter Prüfer, Nicole Schöbi, Alisú Schoua-Glusberg

**Affiliations:** 1 National Center for Health Statistics, Hyattsville, MD, USA; 2 Centre for Comparative Social Surveys, Social Science Building, City University, London, UK; 3 Department of Social Psychology and Methodology, School of Psychology, University of Granada, Spain; 4 RTI International, Research Triangle Park, NC, USA; 5 Agency for Social Analyses (ASA), Sofia, Bulgaria; 6 The National Centre for Social Research, London, UK; 7 Institute of Social Sciences, University of Lisbon, Lisbon, Portugal; 8 GESIS Leibniz Institute for the Social Sciences, Mannheim, Germany; 9 SIDOS, Marin-Epagnier, Switzerland; 10 Research Support Services, Evanston, IL, USA

**Keywords:** cognitive interviewing, comparability, question evaluation, cross-cultural, questionnaire design

## Abstract

This article summarizes the work of the Comparative Cognitive Testing Workgroup, an international coalition of survey methodologists interested in developing an evidence-based methodology for examining the comparability of survey questions within cross-cultural or multinational contexts. To meet this objective, it was necessary to ensure that the cognitive interviewing (CI) method itself did not introduce method bias. Therefore, the workgroup first identified specific characteristics inherent in CI methodology that could undermine the comparability of CI evidence. The group then developed and implemented a protocol addressing those issues. In total, 135 cognitive interviews were conducted by participating countries. Through the process, the group identified various interpretive patterns resulting from sociocultural and language-related differences among countries as well as other patterns of error that would impede comparability of survey data.

The value of cross-national surveys is that they make it possible to examine how different sociocultural, economic, and political systems can impact the material reality of citizens living in those systems. Ironically, however, the very differences that are of interest to cross-national researchers can also impact how individuals in those systems make sense of and respond to survey questions. These structural differences can, in effect, impede a survey’s ability to produce accurate statistics and generate truly meaningful country comparisons of an intended concept. For example, survey data can indicate lower disability rates in particular cultural regions not because there are fewer persons living with physical limitations but because disability is perceived as a shameful condition in those regions and is, therefore, reported less often (Groce 2006).

While a survey’s analytic power can benefit from additional countries to its sample, the addition can also lessen its ability to produce equivalent assessments. This has been a challenge for the European Social Survey (ESS), which has incorporated over 30 countries with diverse cultures and political systems. The entrance of Turkey into the ESS in Round 2 as its first Muslim country, for example, revealed Judaic–Christian assumptions underlying the existing set of religion questions. Similarly, the imported ESS questions on “democracy” generated differing interpretations between the “new” democracies of East and Central Europe and those in Western Europe. Those in new democracies tended to understand the concept as “having free elections,” while those in older democracies saw it as implying “civil rights and liberties” (Fitzgerald and Jowell 2010:488).

The method of cognitive interviewing (CI) can be a particularly useful practice for cross-national surveys because of its potential to identify patterns of interpretation and patterns of error within and across sociocultural and political groups. Because it is qualitative, the method also provides insight into the reason for disparate patterns across groups. For example, a CI study conducted in both poverty-stricken rural Mississippi and the metropolitan Washington, DC, area illustrated that respondents in rural areas with little access to health care were more likely to misunderstand survey questions about various health conditions (i.e., congestive heart failure and COPD) and cancer screening (i.e., mammograms and prostate-specific antigen [PSA] tests). Having adequate health care with providers explaining medical concepts afforded respondents the necessary medical knowledge to understand the survey questions; those without such care did not have the knowledge (Miller 2003). CI can also identify differences in the way that questions have been translated into various languages (Harkness et al. 2003). Additionally, Padilla (2007) demonstrated that CI can provide evidence of “construct overlap,” that is, the extent to which different linguistic and cultural groups understand intended concepts the same way.

The objective of the Comparative Cognitive Testing Workgroup was to develop the methodology as an evidence-based approach for evaluating survey questions within cross-cultural or multinational contexts. A well-conceived CI study should examine the extent to which survey questions work consistently across all countries and subgroups. Specifically, do respondents interpret questions consistently regardless of country, language, or demographic? And, do respondents use the same thought processes to formulate an answer? For this project, the coalition consisted of representatives from the Budapest Initiative (BI), a UNECE/Eurostat/WHO taskforce to develop measures of health states, and the ESS, a biennial cross-national attitude survey conducted in over 30 European countries. (For additional BI information, see www.cdc.gov/nchs/citygroup/products/meeting6/WG6_Session4_1_Madans.ppt. For additional ESS information, see http://www.europeansocialsurvey.org) This coalition consisted of representatives from seven different nations using six languages: the United States (in English and Spanish), the United Kingdom, Bulgaria, Portugal, Switzerland (in French), Germany, and Spain.

In Fall 2007, workgroup members met to discuss project goals and to determine the protocol. Survey questions to be examined by the group included both BI and ESS questions and covered attitudes toward taxes, perceptions of age status, and aspects of physical and mental health. Over the next 5 months, members conducted 135 cognitive interviews in their participating countries, and, in February 2008, held a joint analysis meeting to conduct a systematic, comparative analysis of those interviews. Additional analyses were also conducted after the analysis meeting. This article describes the process developed by the workgroup and illustrates the types of analyses possible as well as the types of findings that can emerge from CI when coordinated across diverse populations and countries.

## Method

To begin the project, a meeting of workgroup members was held in London in September 2007 to lay out the parameters of the project and to establish the testing protocol. Aspects of traditional cognitive testing were discussed and then incorporated into the overall design with adaptation for cross-cultural implementation where required. Those issues included:

1. sample composition, selection, and recruitment;
2. language equivalence and translation procedures;
3. use of nonstandardized probing techniques, the potential impact on data quality and comparability, and the establishment of a semistructured interview guide;
4. differing skill levels of interviewers, potential impact on data quality and comparability, and interviewer training;
5. cognitive interview documentation (i.e., what constitutes a finding and data processing); and
6. analysis of cognitive interview data.

The primary focus of discussion was the issue of what makes a comparable cognitive testing study truly comparable so that it could identify cross-national differences. Indeed, there was a great deal of debate, and differing viewpoints were not always resolved. To this end, we recognize that this project is only a beginning and that more work is required to optimize the method for comparable cross-national investigations.

As would be essential for a survey research project, there was an inclination to suggest standardizing as many aspects of the CI project as possible. However, it was also pointed out that the advantage of CI lies in its interpretive qualities, specifically revealing how and why respondents answered questions the way they did. Furthermore, it was recognized that only through this type of qualitative investigation is it possible to determine the ways and extent to which social and cultural contexts impact the question response process. Therefore, it became important to determine how standardization might also weaken our ability to capture the interpretive processes necessary to perform the comparative analysis. For example, a structured questionnaire was ultimately rejected in favor of a semistructured interviewing protocol whereby interviewers were granted freedom to follow up on emerging themes.

Additionally, given the limited resources, it was important to assess which components would truly contribute to comparability and which components would not. For example, given the small samples in each country, it did not seem necessary or even feasible to require each country to recruit identical samples using the same recruiting procedures. Instead, emphasis of comparability was ultimately placed on data quality, documenting interviews, and performing a joint and coordinated analysis.

Finally, plans were made to ensure communication and coordination across the multiple interviewing locations. Specifically, weekly conference calls were scheduled and time lines were established for making translations and conducting interviews. Additionally, the ESS created a workgroup Web site so that common documents (e.g., the interviewing guide, sample requirements, translation procedures) could be easily accessed and members could pose questions and have discussions with other members of the group. Finally, a final workgroup meeting was scheduled after all interviews were conducted to analyze interview data through a systematic group process. The joint analysis meeting took place in Washington, DC, in February 2008. The following sections detail the design and implementation of the workgroup project.

### Sampling

Countries were asked to conduct a minimum of 10 interviews, although it was agreed that a greater number of interviews would be welcome. It was determined that, while additional interviews would provide richer understanding of response processes in each of the countries, some countries did not have the resources to conduct a larger number. Because qualitative design does not necessitate a one-to-one comparison as it does in quantitative studies, it was decided that an unequal but greater number of interviews in some countries was more advantageous than fewer but equal number in all countries. Samples were to be broadly reflective of different ages, gender, and socioeconomic status. And, to adequately test the BI health state questions, at least half of respondents were to have either a hearing, visual, mobility, or cognitive functioning problem. Since the sample was purposive and based on specific requirements, countries were able to recruit by whatever means were most efficient for them, for example, by placing an advertisement, handing out fliers, or through existing networks of respondents. All countries except Bulgaria provided respondent remuneration of approximately 40 U.S. dollars.

### Data Collection

The interviewing protocol consisted of two sections: a BI component and an ESS component. The interview was semistructured, consisting of the test questions followed by a few general pre-scripted probe questions, for example: “Why did you answer this way?” Interviewers were instructed to spend 30 minutes on each section (BI or ESS), regardless of whether or not that component was completed. Additionally, interviewers were instructed to begin half of their interviews with the BI component and the other half with the ESS questions. The protocol was written in English, with the BI questions posed in U.S. English and the ESS questions posed in British English. Countries conducting interviews in languages other than English were responsible for producing a translated protocol. Countries were required to produce translations using the Translation Review Adjudication Pre-test and Documentation (TRAPD) committee approach (Harkness and Schoua-Glusberg 1998). The country-specific protocols, sample composition, and other project documents can be downloaded from www.europeansocialsurvey.org. The written probes were intended to serve only as a guide for interviewers to illicit how respondents understood the question as well as how they formulated their answer; they were not intended to be used verbatim. During the interview, respondents were asked each survey item and then probed to explain their answer.

Interviewers ranged in their CI experience. Specifically, interviewers for the United States, Spain, Germany, and the United Kingdom were experienced and regularly conducted cognitive testing studies. On the other hand, CI was new to those interviewing for Bulgaria, Portugal, and Switzerland. To compensate for the lack of experience, a training session was held at the London meeting. Particular effort was given to communicate with those newer interviewers throughout the project via conference calls and the ESS Web site.

All interviews were audio-recorded except for those conducted in Spain and U.S. English, which were video-recorded. From these recordings, interviewers wrote detailed sets of notes that were then compiled by question. Interviewers then charted their data in tables formatted to be easily accessible for a thorough joint analysis. The notes from each interview were written in the language of the interview, however, charts were translated into English so that all workgroup members could understand and analyze data across each country.

### Method of Analysis

**Figure 1.**
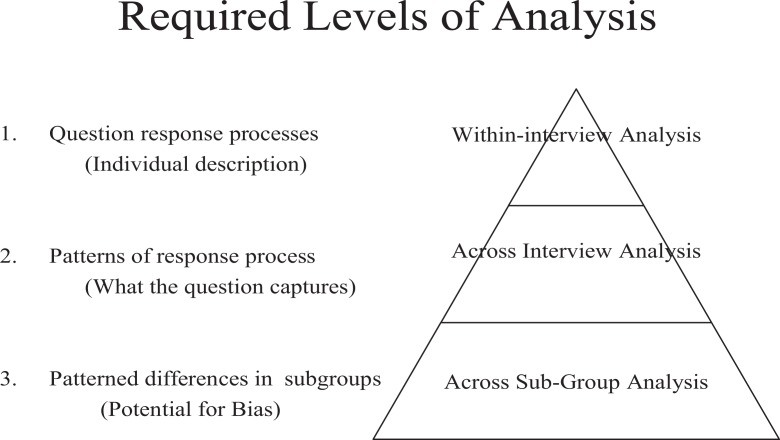
Required levels of analysis for comparative cognitive testing.

To accomplish the research agenda previously outlined, analysis for multinational or cross-subgroup comparative analysis was conceptualized in three distinct layers. Miller (2007) has argued that, to reach conclusions about the comparability of survey questions using cognitive interview data, it is essential to conceptualize analysis in three distinct levels. Figure 1 outlines those levels of analysis.

The first and simplest level of analysis occurs within the interview, specifically, as the interviewer attempts to understand how that respondent has come to interpret, process, and then answer a survey question. The interviewer must act as analyst during the interview, evaluating the information that the respondent describes and following up with additional questions if there are gaps, incongruencies, or disjunctures in the explanation. Additionally, the interviewer must assess the quality and trustworthiness of information being told by the respondent. If the information is deemed problematic, the interviewer must adapt and change interviewing tactics (Rubin and Rubin 2005). Response process errors such as recall trouble or misinterpretation for individual respondents can be identified from this vantage point (i.e., within a single cognitive interview).

The second layer of analysis occurs through a systematic examination of the interviews using a constant comparative method (Glaser and Strauss 1967). Specifically, for each question, interviews should be examined to identify patterns in the way respondents interpret and process the question. By making comparisons across all of the interviews, interpretive patterns can be identified and examined for consistency. If a question asks respondents to evaluate “the system of public benefits and services in their country,” for example, it would be important to understand the degree to which respondents are considering the same types of benefits and services. From this layer of analysis, patterns of calculation across respondents can be identified. This is particularly useful in understanding how qualifying clauses such as “in the past 2 weeks” or “on average” impact the way respondents form an answer, whether respondents consistently use the clause in their calculation, and how inconsistencies might impact reliability of the resulting survey data. At this point, it is possible to identify a theoretical framework for understanding the interpretive meaning behind respondents’ answers and the type of information that is ultimately transported through the survey statistic.

The last level, the heart of the cross-cultural analysis, occurs through examining the various identified patterns across subgroups, determining whether particular groups of respondents are interpreting or processing a question differently. This level of analysis (i.e., identifying patterned differences among subgroups) is particularly important because this is one way in which biased survey data can occur.

To implement these layers of analysis for the comparative workgroup project, cognitive interview data were reduced into relevant constructs (e.g., respondent interpretation, recall accuracy) and charted, allowing an examination across each interview (see Fitzgerald et al. 2009 for chart example). Because the charts were organized by country, subgroup comparisons focused primarily on country and language differences. This was particularly so for analyses conducted in the meeting. In post-meeting analyses, however, when all data for a specific question were combined and examined as a unit by one researcher, it was easier to conduct other types of subgroup comparisons. The remainder of this article illustrates the types of analyses that were performed as well as the types of information gleaned from the joint process (see Miller 2008 for complete analysis and report of BI questions).

## Analysis from the Joint Meeting: Examples from the ESS

The joint analysis meeting was held in Washington, DC, in February 2008. Participants were asked to bring their completed data reduction charts and notes from their interviews as well as bullet points of country-level observations for each ESS question. The bullet-point lists in English provided emerging themes, provisional findings, and the nature of problems experienced by some or all respondents for each of the ESS test questions. The bullets were meant to address the key issues established in the interview protocol and any other emerging issues, such as respondent difficulty answering particular questions or understanding certain terms or concepts, reasons for answering “don’t know,” and evidence suggesting that a question was sensitive or intrusive to respondents. The observations from each country were sent to the ESS team before the meeting to assist with planning the focus of the meeting and served as a starting point for discussion. These preliminary impressions helped structure the analysis meeting and ensured a clear inductive process moving from data to findings.

At the workgroup meeting, analysis consisted of a lead researcher guiding the workgroup through the three levels of the comparative analysis pyramid—first presenting what was identified in the interviews (i.e., the basic errors, interpretations, and ways respondents considered and formulated their answers). Second, the interview data were examined across interviews to determine whether those errors, interpretations, and calculations occurred in patterns. Finally, the patterns were further investigated to determine whether they occurred within a specific subgroup and, if so, the reason for the discrepancy. Charts were used as the primary source of data, but interviewer notes were also referenced when clarification was needed. In a few instances where further clarification was required, workgroup members reviewed recordings of the interviews—though this review occurred after the analysis meeting.

As an interactive process, the joint analysis meeting allowed for the “interrogation” of data, ensuring consistent standards of evidence as well as a systematic and deliberate analysis across countries. Rather than a series of separate and discordant country-level analyses, this was a collective process of comparing interview findings from one data set. And, while the production of a single charted data set in English facilitated a centralized analysis, the joint analysis meeting ensured that the analysis remain steeped in the sociopolitical and cultural contexts of the represented countries. Most importantly, differences discovered across interviews could be more closely scrutinized to determine whether the difference was related to a legitimate comparative issue or was simply an artifact of the method (e.g., differences in country samples, interviewing styles, or level of detail in notes).

This analysis produced insight into the comparability of the survey questions themselves. Specifically, it provided a detailed understanding of question problems and whether or not those problems were related to sociocultural differences, translation problems, or simply generic question design problems.

Significantly, through the three-tiered analytic process, each identified question response problem or interpretive inconsistency could be categorized within a four-item typology of sources of error for multinational survey questions. Based on the experience from questionnaire design in previous rounds of the ESS, the purpose of the typology was to indicate the particular elements of a question or the survey process that could undermine data comparability (Fitzgerald et al. 2009). The components of the typology include: (1) source question problems; (2) translation error; (3) source question and its interaction with translation; and (4) cultural issues. Understanding the specific cause of the problem is important because it provides a foundation for understanding how to manage that particular question design problem. There were occasions in which question problems occurred relatively consistently across each country. That is, the question design was flawed but it was flawed for all countries in a similar way and therefore would likely not specifically undermine cross-cultural comparability. For example, in a question asking about the perceived status of particular age groups, respondents across each country struggled to generalize about an entire age group. In the error typology, Fitzgerald et al. (2009) recognize this type of error as a *source question error*.

On the other hand, country-specific problems did emerge—problems that would undermine comparability if left unattended—and additional investigation was required to explain the discrepancy and, ultimately, to determine how to amend the question. In some instances, a country-specific problem was related to the particular translation. For example, only through the discovery of differing interpretations in the Portuguese interviews and the ensuing discussion with the Portuguese researcher was it discovered that the particular translation of the question asking about the income tax authorities had omitted any reference to income tax. Fitzgerald et al. (2009) specifically describe *translation error* as an error originating from either a genuine human mistake or an inappropriate choice of translation. Once discovered, this type of translation problem can be easily corrected.

Fitzgerald et al. (2009) distinguish this type of error, however, from the typology’s third category (*source question and its interaction with translation*) because the translation problem is not a simple human error and is not easily corrected. In this category, although a question may work well in the source language, an intrinsic aspect of the question’s design makes translation difficult or especially burdensome, increasing the chance of error. In the ESS testing, for example, a particular question assessed how likely it was that respondents thought people under the age of 30 could be described as”moral.” Discussion in the joint analysis meeting suggested that the direct translation of moral was not an often-used word in everyday language. Consequently, finding a functionally equivalent term or phrase was a challenge, and the translations that were ultimately chosen resulted in comparability problems. This caused a particular problem in Germany where respondents did not consider morality to be the notion of “knowing right from wrong,” as was considered by other countries’ respondents. In the end, although large differences were identified in only one country, the joint analysis meeting concluded that the potential for additional comparability problems in other countries in the ESS at a later stage ultimately determined that the word “moral” would not be used.

Finally, Fitzgerald et al. (2009) describe the last source of error (*cultural error*) as being unrelated to translation, but rather the concepts—outside of the language—that either do not exist in each country or do not exist in a form similar enough to allow equivalent measurement. This would also include circumstances whereby concepts have such varied salience between countries that it creates measurement difficulty. For example, the joint analysis suggested that an ESS question asking respondents to identify their preference among three different tax collection systems might not be comparable for those Swiss respondents who, compared to all the other countries, appeared to have little knowledge of tax systems on which to base their answer. Although knowledge of the tax system was not required to answer this question, some respondents reported that they felt this was needed. Upon further discussion, it was suggested by the Swiss researcher that because responsibility for complete household level tax returns is the sole responsibility of the head of the household, other household members might be unwilling to give an answer or alternatively might provide a nonattitude.

## Post-Meeting Analysis: Examples from the Budapest Initiative

Because of limited time, much of the analysis of the BI questions could not be completed in the joint meeting, and the remaining analysis occurred after the meeting, with one researcher analyzing the charts and then following up with group members for clarification. (Again, see Miller 2008 for the entire BI cognitive testing report.) While less than ideal because team members could not readily provide clarification or pose informed theories about their country’s respondents, conducting an analysis with the entire data set allowed for greater flexibility as to the kinds of analyses that could be conducted and maximized the potential for discovery. Specifically, questions could be compared across other questions to identify subtle pattern differences, and, as previously noted, subgroup comparisons no longer had to be restricted to across-country comparisons. This more complex type of analysis was simply not feasible in the meeting. The BI questions about pain is a good illustration of this type of analysis.

A primary goal of the BI testing project was to study how, within the context of survey questions, respondents conceptualize and report on pain. Because experiences of pain involve multiple factors (e.g., frequency, intensity, time period, impact, and seriousness of condition), designing one or two questions that capture respondents’ full experience is difficult. And, because respondents have many paths by which they can conceptualize, calculate, and formulate a summary indicator, developing a measure that achieves validity and reliability, as well as comparability across subgroups, is challenging.

Within the BI taskforce, there were differing approaches to question design for pain. The first theorized that, because it is impossible to account for all of the possible dimensions of pain, it is best to ask for one composite score requiring respondents to average their pain experiences across a week’s time. This was the intent of the first pain question, Pain 1 (see Box 1).

Box 1 Summary Pain Measure

Pain 1: Overall, during the past week, how much physical pain or discomfort did you have? Would you say: none at all, a little, moderate, a lot, or extreme physical pain or physical discomfort?

The second approach contended that, because bouts of pain can fluctuate dramatically across a week, it would be conceptually difficult for respondents to report one composite and meaningful score. Not specifying these constructs, as Pain 1 does, could lead to questionable validity and reliability of the measure. Therefore, the intent of the next questions, Pain 2a and Pain 2b, was to disentangle two components of pain, asking respondents to consider and report the number of days and intensity separately (see Box 2). An important part of the analysis, then, was to examine how these two designs compared and whether there were any differences across countries.

Box 2 Disentangled Pain Measure

Pain 2a: How many days during the past week did you have physical pain or discomfort?

Pain 2b: During those times when you had physical pain or discomfort, how would you describe your level of physical pain or discomfort? Would you say it was mild, moderate, severe or extreme?

In the joint meeting with country participants overseeing the data from their own interviews, it was possible to examine interpretations of pain and to make across-country comparisons. For example, in all countries, respondents considered a broad range of painful conditions or experiences, including arthritis, a bad fall, a root canal, and sore muscles from exercise—essentially including any incident or episode that was perceived as painful. Regarding the amount or magnitude of respondents’ pain, it was difficult for respondents to explain in detail how they arrived at their answer. Other than simply describing their pain in terms of “it was a lot” or “it was very bad,” respondents were limited in their ability to speak to the actual intensity of their pain. Instead, to explain their answer, respondents either referenced the impact of the pain on their lives, their need for or use of pain medication, or the concern they had that the pain indicated a serious health problem. This was also consistent across countries.

Discussion from the joint analysis meeting also revealed that a translation choice regarding the word “discomfort” created a potential comparability problem. Depending on the word chosen for the translation, discomfort could mean a lower threshold of pain (which is the intended interpretation) or a general sense of “uncomfortableness” (which is not the intended interpretation). For example, one Bulgarian respondent stated that discomfort occurs after eating or drinking too much and getting no sleep, whereas pain is a much graver situation.

Because pain intensity is a uniquely subjective phenomenon, it was impossible in the interview to investigate the validity of each respondent’s answer. Even by examining the way respondents justified their answer, it was impossible to determine the accuracy of their response. Instead of considering perceptions of pain and the inevitable variability of those perceptions, the central concern became understanding how respondents arrived at their answer, specifically, the various factors they considered and the calculations that they performed as well as how consistently they would perform those calculations. Examining how respondents answered Pain 1 in relationship to Pain 2, as illustrated in Table 1, provides a unique window into the performance of the questions because it clarifies inconsistencies in respondents’ reports.

**Table 1.** Respondents Reports for Pain 1 by Pain 2a and b

		Pain 1: Summary Measure				
		None	A little	Moderate	A lot	Extreme
Pain 2a and b: Disentangled Measure			SW11			
Mild intensity	1–2 Days	USE24	**USE28 B1GB27 GB32 USS4 S14 S16 G5 B2**	S17 P3		
	3–5 Days		**S03 B4 GB47 USE34 GB15**			
	6–7 Days		**SW14 S04 G9 GB21**	USE27 USS7 S11		
Moderate intensity	1–2 Days	S09 P4	S15 S05 S12	**GB12 GB34**	USE14	B7
	3–5 Days		USE38 GB24 USE18 G3	**USE15 GB24 USE12 SW3 SW6 B8**		
	6–7 Days		USS6 S07 S08 GB43	**SW23 GB45 S13 S06 G2 GB26**		
Severe intensity	1–2 Days		G10	SW24 GB36		
	3–5 Days					
	6–7 Days		GB13	B9	**USE26 USS3 SW34 P6 B5 GB11GB41GB25**	
Extreme intensity	1–2 Days					**GB28**
	3–5 Days					
	6–7 Days			USE23		**SW22**

Note: The letters code indicates country of interview: USE = United States English version, USS = United States Spanish version, SW = Switzerland, P = Portugal, G = Germany, B = Bulgaria, GB = Great Britain, and S = Spain. The number indicates the interview identification of the country.

The letter–number combinations appearing in the cells are the specific identifications for individual respondents within countries: USE—U.S. English, USS—U.S. Spanish, Sw—Switzerland, P—Portugal, G—Germany, B—Bulgaria, GB—Great Britain, and S—Spain. The identifications in the shaded cells represent those respondents whose answers to the three questions were not consistent, that is, their answers to Pain 1 do not correspond to their answer to Pain 2a and b. Of the 68 respondents who were asked all three of the questions, 19 respondents (a full 28%) gave relatively inconsistent answers. Interestingly, 62% of all the respondents gave the exact same response in Pain 2 as they did in Pain 1 (those cases in bold type), and those cases appear almost equally across the number of days for each response category. Consequently, it appears that for Pain 1, these respondents did not average their pain across the entire week as required by the question. Instead, they likely made a rough estimate or, if they did compute a score, did not calculate across the entire week.

It is also important to note the rather high frequency of moderate responses. Of the 66 respondents, two-thirds answered “moderate” to at least one of the questions. Almost 25% of respondents answered moderate to both questions; 35% answered moderate to the first, and 44% answered moderate to the second. This suggests, again, that respondents are grossly estimating rather than accurately assessing and performing the calculation. Hence, with the combination of Pain 2a and Pain 2b, relatively more detailed and accurate information can be collected regarding the nature of the pain than with Pain 1 alone. For example, in Pain 1, those respondents who reported only one day of mild pain are considered the same as those who reported mild pain every day. The combination of Pain 2a, b makes a distinction between these types of cases.

Many of the inconsistencies are indicative of how respondents report on pain, specifically, how they can consider different dimensions of pain and use varied methods in constructing an answer each time they answer a question (even, as in this case, when the questions occur consecutively). Examining the inconsistent cases provides insight into the instability of reports on pain, particularly how context and idiosyncratic response processes can dramatically affect an answer. For example, two of the respondents who answered “none” in Pain 1 but reported pain in Pain 2a and 2b; both answered none to Pain 1, thinking that pain medicine alleviated their head- or backache, but moments later based their answers to Pain 2b on the level of pain prior to taking the pain reliever. Conversely, a Bulgarian respondent who, in Pain 1, reported extreme pain and stated that “pills keep me alive,” answered “moderate” to Pain 2b—explaining that the medication manages the pain, so it is not so intense. In another inconsistent case, a Spanish respondent explained that he pulled a muscle when working out, but when answering Pain 1 did not believe it was serious enough to report. By the next pain question (for whatever reason), he changed his mind and reported having “moderate” pain on at least one day. Similarly, in answering Pain 1, a British respondent considered his mild arthritis and a kink in his neck from a bad pillow and, consequently, answered “a little.” However, by Pain 2b, he remembered a terrible headache that he had a few days before and, therefore, reported “severe.”

## Conclusion

The main objective of this project was to develop CI methodology such that it could be used as an evidence-based method for examining the comparability of survey questions within cross-cultural or multinational contexts. By employing a comparative analysis of cognitive interview data, the method can provide insight into whether a particular pattern might be idiosyncratic or could produce systematic bias in the resulting survey data. A well-conceived CI study, therefore, within this cross-cultural context, should examine the extent to which the survey questions work consistently across all countries and subgroups. Contingent on addressing these comparative issues, explanations for the inconsistencies should emerge simultaneously. That is, the study should be able to determine whether those differences reflect true sociocultural differences across the groups or whether they reflect a translation or wording problem in a particular country. It is also possible that differences are merely artifacts of the CI study itself, for example, if one country interviews only highly educated respondents.

To design such a study, it is essential to consider key aspects of CI methodology. Those aspects include: sample composition, selection and recruitment, language equivalence and translation procedures, use of nonstandardized probing techniques, differing skill levels of interviewers, data quality and comparability, what constitutes a finding, and cognitive interview documentation. Additionally, it is critical to conduct a thoughtful multilayer analysis that is systematic and evidence based. With this type of insight, a study should be able to indicate how to fix or manage these differences through question design. Successfully accomplishing these goals through a CI study prior to fielding allows survey designers the opportunity to improve equivalence or to provide documentation regarding the appropriate interpretation of the survey data.

A number of significant challenges emerged during this project, including the expense of sending participating researchers to two meetings and the high work load associated with charting and analyzing data. There was also not enough time in the 3-day meeting to cover all of the questions. In hindsight, a more elaborate analysis was needed for the BI component prior to the joint analysis. In the future, this level of analysis would be carried out before the joint meeting. Finally, converting the interviews into charted data in English posed an extra burden for countries in which English was not their native language. This, however, proved to be invaluable in that the process created a single CI data set that was accessible to all researchers working on the project.
